# Risk factors for mortality in TB patients: a 10-year electronic record review in a South African province

**DOI:** 10.1186/s12889-016-3972-2

**Published:** 2017-01-06

**Authors:** J Christo Heunis, N Gladys Kigozi, Perpetual Chikobvu, Sonja Botha, HCJ Dingie van Rensburg

**Affiliations:** 1 Centre for Health Systems Research and Development, University of the Free State, P.O. Box 399, Bloemfontein, 9300 South Africa; 2 Free State Department of Health, P.O. Box 277, Bloemfontein, 9300 South Africa; 3 Department of Community Health, University of the Free State, P.O. Box 399, Bloemfontein, 9300 South Africa; 4 JPS Africa, Postnet Suite 132, Private Bag X14, Brooklyn, Pretoria 0011 South Africa

**Keywords:** Tuberculosis, Mortality, Risk factors, Free State Province, South Africa

## Abstract

**Background:**

Since 1990, reduction of tuberculosis (TB) mortality has been lower in South Africa than in other high-burden countries in Africa. This research investigated the influence of routinely captured demographic and clinical or programme variables on death in TB patients in the Free State Province.

**Methods:**

A retrospective review of case information captured in the Electronic TB register (ETR.net) over the years 2003 to 2012 was conducted. Extracted data were subjected to descriptive and logistic regression analyses. The outcome variable was defined as all registered TB cases with ‘died’ as the recorded outcome. The variables associated with increased or decreased odds of dying in TB patients were established. The univariate and adjusted odds ratios (OR and AOR) together with their corresponding 95% confidence intervals (CI) were estimated, taking the clustering effect of the districts into account.

**Results:**

Of the 190,472 TB cases included in the analysis, 30,991 (16.3%) had ‘died’ as the recorded treatment outcome. The proportion of TB patients that died increased from 15.1% in 2003 to 17.8% in 2009, before declining to 15.4% in 2012. The odds of dying was incrementally higher in the older age groups: 8–17 years (AOR: 2.0; CI: 1.5–2.7), 18–49 years (AOR: 5.8; CI: 4.0–8.4), 50–64 years (AOR: 7.7; CI: 4.6–12.7), and ≥65 years (AOR: 14.4; CI: 10.3–20.2). Other factors associated with increased odds of mortality included: HIV co-infection (males – AOR: 2.4; CI: 2.1–2.8; females – AOR: 1.9; CI: 1.7–2.1) or unknown HIV status (males – AOR: 2.8; CI: 2.5–3.1; females – AOR: 2.4; CI: 2.2–2.6), having a negative (AOR: 1.4; CI: 1.3–1.6) or a missing (AOR: 2.1; CI: 1.4–3.2) pre-treatment sputum smear result, and being a retreatment case (AOR: 1.3; CI: 1.2–1.4).

**Conclusions:**

Although mortality in TB patients in the Free State has been falling since 2009, it remained high at more than 15% in 2012. Appropriately targeted treatment and care for the identified high-risk groups could be considered.

## Background

Numbers of deaths and concomitant mortality rates represent traditional measures of the burden and impact of diseases and the state of public health [1]. Tuberculosis (TB) ranks among the ten principal causes of death and disability worldwide [2]. South Africa has one of the world’s most serious TB epidemics that in recent decades has been driven by the human immunodeficiency virus (HIV) epidemic [3]. In 2015, among the 22 countries with the highest burden of TB, the country had the fourth highest estimated incidence of TB and the highest number of HIV-infected TB cases and deaths [4].

While South Africa has made notable progress in reducing TB prevalence and deaths and improving treatment outcomes for new smear-positive TB cases [5], overall reduction of TB mortality of only 6% over the years 1990 to 2013 is substandard considering that over the same period TB deaths declined by 45% globally and 40% in the African region [6]. The World Health Organization’s (WHO) ‘End TB Strategy’ has set the target to reduce TB deaths by 95% by the year 2035. In order to reach this target, the proportion of people with TB who die from the disease should decline from a projected 15% in 2015 to 6.5% by 2025 [7].

TB and HIV control efforts in South Africa are mainly driven by the public health sector following a district-based primary health care (PHC) approach and provision of free treatment and care services. Located in the centre of South Africa bordering on Lesotho, the Free State has an estimated population of 2.8 million [8]. TB was the leading underlying cause of death in the province in 2013 [9]. In 2012, of the nine provinces it reported the fifth highest TB incidence at 709 cases per 100,000 population, the second highest TB death rate at 142 cases per 100,000 population, and the third highest prevalence of antenatal HIV infection at 32.1% [10].

Understanding the factors leading to death following TB diagnosis is important for prognostic purposes, but also for programme planning and appropriate targeting of care to high-risk groups. The study set out to establish the influence of routinely captured demographic and clinical or programme variables on mortality in TB patients in the Free State over the years, 2003–2012.

## Methods

### Setting and design

A retrospective record review of individual case information captured in the Electronic TB register (ETR.net) was conducted. Based on the WHO and International Union Against Tuberculosis and Lung Disease (IUTLD) format of recording and reporting, the electronic format of TB case information collection and collation is widely recognised as a valuable tool to gather and analyse TB and TB-HIV surveillance data in order to monitor and evaluate programme performance. The ETR.net was implemented in South Africa in 2003. In the Free State, TB case information is processed as follows: firstly, at the primary healthcare facility level, data are recorded in a paper-based treatment register; secondly, at the subdistrict level, captured on the ETR.net; and, finally, at the provincial level, aggregated for the province as a whole [11].

### Population, data management and measurements

**Fig. 1:**
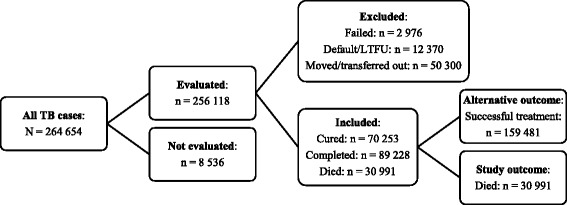
Overview of TB cases selected for the study

The study population was defined as all cases in the provincial surveillance population, i.e. all cases registered in the ETR.net, over the years 2003 to 2012. Duplicate entries were deleted and patient names removed before data were extracted from the database. The outcome variable was defined as all patients with ‘died’ – as compared to ‘successful treatment’ (‘cured’ or ‘completed’) – as the recorded outcome (Fig. 1). Cases with ‘not evaluated’, ‘failed’, ‘defaulted/LTFU’, ‘moved/transferred out’ as the recorded outcome were excluded from the analysis.

**Fig. 2:**
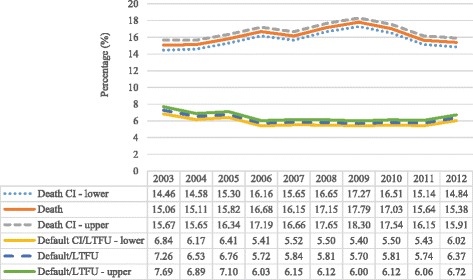
Death and default/LTFU in TB patients, 2003–2012

Although the outcome ‘loss to follow up’ (LTFU) has been associated with TB mortality [12, 13], it could not be included in the current study due to the high patient mobility and lack of unique patient identifiers and inter-linked ETR.net databases across facilities and provinces. This meant that TB programme staff could not trace patients to determine if they had taken up treatment at another facility in the same or another district or province and ‘LTFU’ cases were probably recorded as ‘defaulted’. However, a separate analysis was conducted where the default/LTFU cases were considered to have died. The estimated odds ratios were then compared to those when the defaulters/LTFU cases were excluded. There were no statistically significant differences in the estimated odds ratios except in the age group ≥65 years where the odds ratio significantly decreased (Table 1). Yet only 145 of the 12,370 cases with default/LTFU as the recorded outcome represented this age group. Also, compared to the fluctuating rate of cases recorded as died over the study period, the trend in cases recorded as default/LTFU remained relatively stable (Fig. 2). Thus, the exclusion of the default/LTFU cases from the final analysis implied minimal bias. The study also excluded drug-resistant cases as they are not recorded in the basic ETR.net, but in a separate Electronic Drug-Resistant Tuberculosis Register (EDRWeb.net).

**Table 1:** Risk factors for death in TB patients

Variables		*N* = 30 991 n (%)	Univariate results OR (95% CI)	Full adjusted model – died and default/LTFU AOR (95% CI)	Full adjusted model – died only AOR (95% CI)	Males AOR (95% CI)	Females AOR (95% CI)
Gender	Female (Ref)	13 834 (44.64)	1	1	1	-	-
	Male	17 157 (55.30)	1.0 (0.9–1.2)	0.8 (0.6–1.0)	0.8 (0.7–0.9)	-	-
Age (years)	≤7 (Ref)	943 (3.04)	1	1	1	1	1
	8–17	472 (1.52)	1.4 (1.2–1.6)	1.9 (1.6–2.3)	2.0 (1.5–2.7)	2.0 (1.3–3.2)	2.0 (1.8–2.2)
	18–49	23 266 (75.07)	4.1 (3.8–4.5)	4.0 (3.0–5.4)	5.8 (4.0–8.4)	6.4 (4.5–9.0)	5.3 (3.6–7.9)
	50–64	5 094 (16.44)	5.2 (4.1–6.6)	4.4 (2.9–6.5)	7.7 (4.6–12.7)	9.0 (6.0–13.4)	6.8 (3.9–12.1)
	≥65	1 216 (3.92)	8.4 (7.2–9.9)	7.5 (6.1–9.2)	14.4 (10.3–20.2)	13.8 (10.1–18.9)	14.4 (9.9–21.0)
HIV status	Negative (Ref)	1 787 (5.77)	1	1	1	1	1
	Positive	10 748 (34.68)	2.2 (2.0–2.5)	1.6 (1.4–1.9)	1.8 (1.6–2.1)	2.4 (2.1–2.8)	1.9 (1.7–2.1)
	Unknown	18 456 (59.55)	2.8 (2.6–3.0)	1.9 (1.7–2.1)	2.4 (2.2–2.5)	2.8 (2.5–3.1)	2.4 (2.2–2.6)
Pre-treatment sputum smear result	Smear + (Ref)	12 047 (38.87)	1	1	1	1	1
	Smear -	7 375 (23.80)	1.5 (1.4–1.7)	1.3 (1.2–1.5)	1.4 (1.3–1.6)	1.4 (1.2–1.6)	1.5 (1.3–1.6)
	No result	11 569 (37.33)	1.6 (1.4–1.8)	1.9 (1.4–2.6)	2.1 (1.4–3.2)	2.3 (1.5–3.4)	2.0 (1.3–3.1)
Treatment category	New (Ref)	24 587 (79.34)	1	1	1	1	1
	Retreatment	6 404 (20.66)	1.4 (1.3–1.5)	1.4 (1.3–1.5)	1.3 (1.2–1.4)	1.3 (1.2–1.4)	1.3 (1.2–1.4)
Disease classification	Both (Ref)	776 (2.50)	1	1	1	1	1
	PTB	23 210 (74.89)	1.3 (1.0–1.5)	1.0 (1.0–1.1)	1.0 (0.9–1.1)	1.0 (0.8–1.2)	1.0 (0.7–1.4)
	EPTB	7 005 (22.60)	0.8 (0.6–0.9)	1.1 (0.9–1.3)	1.0 (0.8–1.2)	1.0 (0.8–1.1)	1.0 (0.7–1.3)
Treatment delay	≤14 days (Ref)	12 820 (41.37)	1	1	1	1	1
	>14 days	3 244 (10.47)	1.0 (0.9–1.1)	0.9 (0.9–1.0)	0.9 (0.9–1.0)	0.9 (0.8–1.0)	0.9 (0.9–1.0)
	Missing	14 927 (48.17)	1.4 (1.3–1.5)	1.1 (0.9–1.2)	1.0 (0.9–1.2)	1.0 (0.9–1.1)	1.1 (0.9–1.3)
HIV status x gender^a^	Male x HIV positive	-	-	1.23 (1.15–1.32)	1.33 (1.26–1.41)	-	-
	Male x HIV unknown	-	-	1.16 (1.09–1.25)	1.20 (1.15–1.25)	-	-

OR odds ratio; AOR adjusted odds ratio; CI confidence interval

^a^Adjusted for HIV status, gender and HIV status x gender interaction

Two demographic variables were included in the analysis: 1) age (≤7 or 8–17 or 18–49 or 50–64 or ≥65 years) and 2) gender (male or female). Five clinical or programme variables that were significantly related to the study outcome in univariate analysis were included in the multivariate models: 1) HIV status (negative or positive or unknown), 2) pre-treatment sputum smear result (positive or negative or no result), 3) treatment category (new or retreatment), 4) disease classification (pulmonary TB [PTB] or extrapulmonary [EPTB] or both), and 5) treatment delay (≤14 days or >14 days after notification).

### Analysis

All data were analysed using STATA 12 [14]. Percentage was used to describe the mortality trend over the study period. Association between the independent variables and treatment outcome was established using the Pearson’s *χ* ^2^ test. Logistic regression analysis was conducted to ascertain factors associated with death in TB patients taking into account the clustering effect of the districts. The univariate and multivariate adjusted odds ratios (OR and AOR) together with their corresponding 95% confidence intervals (CI) were estimated. The assumed significance level was 0.05. During the development of the adjusted model an assessment was carried out to determine if gender was significantly involved in interactions with any of the other independent variables. There was a significant interaction between gender and HIV status which was taken into consideration in presenting results for the adjusted model (Table 1).

### Ethical considerations

Ethical clearance was granted by the Ethics Committee of the Faculty of Health Sciences, University of the Free State (ECUFS 186/2014). Given that the case information was aggregated and anonymised at the provincial level before analysis, a waiver for individual patient consent was granted.

## Results

### Mortality trend

A total of 190,472 cases were included in the study with 16.3% recorded as ‘died’ (Fig. 1). Mortality among TB patients increased from 15.1% in 2003 to 17.8% in 2009 before declining to 15.4% in 2012 (Fig. 2).

Table 1 depicts the demographic and clinical or programme risk factors for death in TB patients.

### Demographic variables

More than half (55.3%) of the patients who died were males. Gender was not independently associated with mortality (OR: 1.0; CI: 0.9–1.2), however, after adjusting for other variables in the model, the risk of dying among males was significantly lower compared to females (AOR: 0.8; CI: 0.7–0.9). The large majority (*n* = 23,266; 75.1%) of TB patients that died were aged 18–49 years. Advancing age was independently associated with death in TB patients and the strength of this association increased after adjusting for other variables in the model. Compared to those in the 0–7 years age group, the odds of death increased from 2.0 (CI: 1.5–2.7) in the 8–17 year old group to 14.4 (CI: 10.3–20.2) in the ≥65 year old group. Table 1 shows that the odds of mortality was higher for males compared to females in the age group 18–49 years – males (AOR: 6.4; CI: 4.5–9.0) and females (AOR: 5.3; CI: 3.6–7.9) and in the age group 50–64 years – males (AOR: 9.0; CI: 6.0–13.4) and females (AOR: 6.8; CI: 3.9–12.1). The odds of death was higher for females (AOR: 14.4: CI 9.9–21.0) in the age group >65 years than for males (AOR: 13.8: CI 10.1–18.9).

### Clinical or programme variables

HIV co-infection (OR: 2.2; CI: 2.0–2.5) and unknown HIV status (OR: 2.8; CI: 2.6–3.0) were independently associated with increased likelihood of death while on TB treatment. HIV status significantly interacted with gender and stratified results indicate that the risk of death was higher among both male (AOR: 2.4; CI: 2.1–2.8) and female (AOR: 1.9; CI: 1.7–2.1) HIV-infected cases compared to their respective HIV-uninfected counterparts. Likewise, both male (AOR: 2.8; CI: 2.5–3.1) and female (AOR: 2.4; CI: 2.2–2.6) TB cases with unknown HIV status had higher risk of dying compared to their HIV-uninfected counterparts. Having a negative (AOR: 1.4; CI: 1.3–1.6) or a missing (AOR: 2.1; CI: 1.4–3.2) pre-treatment sputum smear result was significantly associated with increased risk for death in univariate analysis, with the risk increasing after controlling for other factors included in the model. Patients categorised as retreatment cases had significantly higher risk of death in univariate analysis (OR: 1.4; CI: 1.3–1.5) and only slightly lesser so in multivariate analysis (AOR: 1.3; CI: 1.2–1.4). Independently, EPTB disease classification (OR: 0.8; CI: 0.6–0.9), as compared to having both PTB and EPTB, was significantly protective against death, but the association became insignificant after adjusting for other factors in the model.

## Discussion

The record review in the Free State showed that mortality in TB patients increased from 15.1% in 2003 to 17.8% in 2009, before declining to 15.4% in 2012, thus a net 10-year increase of 0.3%. The steady rise in mortality in TB patients over the years 2003 to 2009 is variously attributed to migration [15] and high levels of HIV co-infected multidrug-resistant TB [16]. The marked decline in the mortality trend after 2009 can possibly be attributed to the progressive evolving of clinical eligibility requirements for HIV co-infected TB cases to access antiretroviral treatment (ART) [17], as well as heightened political attention and international support and civil society mobilisation around TB and HIV, including the launch of a national HIV/TB campaign in 2011 [4, 18].

The record review in the Free State showed that TB cases in older age groups had significantly increased odds of death. Among the variables included in the unadjusted and adjusted regression models, advancing age was the strongest risk factor for mortality in TB patients. Compared to cases aged 0–7 years, risk for death was incrementally higher in each older age group. The greatest proportion of deaths occurred among those aged 18–49 years. This corresponds to research in Limpopo [19] and Western Cape [20] (South Africa) that associated higher mortality in TB patients with advancing age. An international study observed that although TB age distributions in Africa have been severely skewed by the HIV epidemic, disease burdens among older people are increasing [21]. According to this study, older adults are more likely to develop extrapulmonary and atypical forms of TB that are often hard to diagnose. Older patients’ treatment and care is also complicated by more frequent drug-related adverse events and increased co-morbidity.

When adjusting for other factors, male gender was significantly protective against death in TB patients in the current study. This corresponds with the findings of research in Western Cape that associated female gender with increased risk of death in HIV-infected TB cases [20]. The WHO reports that although globally the numbers of HIV-associated TB deaths were similar among men and women in 2011, more deaths were estimated to have occurred among women than men in the African region, whilst in other regions more deaths were estimated to have occurred in men [22]. The same source states that TB in pregnant women living with HIV increases the risk of maternal mortality by almost 300% and that in Africa TB rates are about ten times higher among pregnant women living with HIV than in their HIV uninfected counterparts. A systematic review of qualitative studies to identify gender-related barriers in accessing TB diagnostic and treatment services showed that women experience financial dependence, lower general literacy and household stigma as barriers [23].

After advancing age, being co-infected with HIV or having unknown HIV status was the strongest risk factor for increased mortality in TB patients. However, for the period under study, 2003 to 2012, the HIV status of the majority (59.6%) of cases that died while on TB treatment was unknown. Since integrated TB-HIV data gathering in the ETR.net in the Free State started only in 2009, it was not possible to measure the effect of ART and varying CD4 count levels on mortality in TB cases for the whole study period. Recent research in Swaziland revealed that high mortality in TB-HIV patients has persisted despite increasing ART coverage in TB patients [24]. A separate study is in process to measure the effect of ART in TB-HIV co-infected cases in the Free State for the years 2009 to 2012.

Not having a pre-treatment sputum smear result was the third strongest risk factor for death in TB patients. In KwaZulu-Natal (South Africa), research found that over one-third of registered TB cases could not be confirmed based on laboratory results [25]. Many cases did not have a laboratory record, lending to uncertainty about the validity of the smear results and treatment outcomes recorded in the ETR.net.

Having a negative pre-treatment sputum smear result was the fourth strongest risk factor for death in TB patients. Research in Malawi [26] and Zimbabwe [27] established that smear-negative PTB patients presenting with signs of concurrent HIV infection were at particularly high risk of death. A systematic review has explained that in regions of high HIV prevalence poor outcomes of smear-negative TB is a feature of advancing immunosuppression and therefore higher risk of death from other AIDS-related disease when compared to the more robust immunological profile in patients with smear-positive TB [28].

Being a retreatment case was the fifth strongest risk factor for dying while on TB treatment. This is corroborated by similar research findings related to patients with previous episodes of TB in Limpopo (South Africa) [19], Uganda [29] and Malaysia [30]. In 2014, 6.7% of retreatment as opposed to 1.8% of new cases in South Africa had multidrug-resistant TB [4]. During most of the study period, 2003–2012, culture was the main tool for drug-susceptibility testing which implied a need for trained personnel and expensive laboratory equipment and long lengths of time to receive results while in the interim patients were commonly commenced on drug-sensitive TB regimens. The number of Xpert MTB/RIF tests that were conducted in the province has since increased from 1,199 in 2012 to 6,734 in 2013 and 8,062 in 2014 [31]. Expansion of the use of Xpert MTB/RIF is expected to have a substantial overall TB mortality reduction effect in Africa if wide population coverage can be achieved [32]. Further research may be necessary to understand its influence on the outcomes of retreatment cases specifically. Xpert MTB/RIF is also an effective method to diagnose smear-negative TB [33].

Most surveillance and notification systems are affected by a degree of underestimation and therefore uncertainty surrounds the 'true’ incidence of disease affecting morbidity and mortality rates [34]. Electronic reviews are also limited in the range of variables that can be included for purposes of explaining risk for mortality among TB patients. The register reflects only all-cause mortality; it does not specify whether the death was actually TB-related. Another limitation of study is that since integrated TB-HIV data gathering in the ETR.net in the Free State started only in 2009, it was not possible to measure the protective effect of HIV-related clinical interventions in TB-HIV co-infected patients for the study period, 2003–2012.

## Conclusions

Although the mortality among TB patients decreased over the years 2009–2012, it remained high at more than 15%. The factors increasing the odds of death in TB patients included advancing age, female gender, HIV co-infection or unknown HIV status, having a negative or a missing pre-treatment sputum smear result, and being a retreatment patient. Appropriate targeting of care to these high-risk groups could be considered.
